# Cold Atmospheric Plasma (CAP) Changes Gene Expression of Key Molecules of the Wound Healing Machinery and Improves Wound Healing *In Vitro* and *In Vivo*

**DOI:** 10.1371/journal.pone.0079325

**Published:** 2013-11-12

**Authors:** Stephanie Arndt, Petra Unger, Eva Wacker, Tetsuji Shimizu, Julia Heinlin, Yang-Fang Li, Hubertus M. Thomas, Gregor E. Morfill, Julia L. Zimmermann, Anja-Katrin Bosserhoff, Sigrid Karrer

**Affiliations:** 1 Institute of Pathology, University Regensburg, Regensburg, Germany; 2 Department of Dermatology, University Hospital Regensburg, Regensburg, Germany; 3 Max-Planck-Institute for Extraterrestrial Physics, Garching, Germany; MGH, MMS, United States of America

## Abstract

Cold atmospheric plasma (CAP) has the potential to interact with tissue or cells leading to fast, painless and efficient disinfection and furthermore has positive effects on wound healing and tissue regeneration. For clinical implementation it is necessary to examine how CAP improves wound healing and which molecular changes occur after the CAP treatment. In the present study we used the second generation MicroPlaSter ß® in analogy to the current clinical standard (2 min treatment time) in order to determine molecular changes induced by CAP using *in vitro* cell culture studies with human fibroblasts and an *in vivo* mouse skin wound healing model. Our *in vitro* analysis revealed that the CAP treatment induces the expression of important key genes crucial for the wound healing response like IL-6, IL-8, MCP-1, TGF-ß1, TGF-ß2, and promotes the production of collagen type I and alpha-SMA. Scratch wound healing assays showed improved cell migration, whereas cell proliferation analyzed by XTT method, and the apoptotic machinery analyzed by protein array technology, was not altered by CAP in dermal fibroblasts. An *in vivo* wound healing model confirmed that the CAP treatment affects above mentioned genes involved in wound healing, tissue injury and repair. Additionally, we observed that the CAP treatment improves wound healing in mice, no relevant side effects were detected. We suggest that improved wound healing might be due to the activation of a specified panel of cytokines and growth factors by CAP. In summary, our *in vitro* human and *in vivo* animal data suggest that the 2 min treatment with the MicroPlaSter ß® is an effective technique for activating wound healing relevant molecules in dermal fibroblasts leading to improved wound healing, whereas the mechanisms which contribute to these observed effects have to be further investigated.

## Introduction

Cold atmospheric plasma (CAP) is a partly ionized gas – produced at room temperature and atmospheric pressure - which is created by electric discharges. The usage of CAP in therapy has developed into an innovative field in medicine in the last years – especially because CAPs can be applied contact-free without inducing pain. One possible application in therapy – the treatment of wounds – attracted a lot of interest: In randomized clinical studies CAP proved to reduce bacteria in chronic wounds – independent of the present species and their resistance level - and to promote wound healing [1]–[3]. In contrast to ointments, CAP – as a gas - can access even microscopic openings and ragged surfaces. Another great advantage of this physical method is, that in contrast to antibiotic or antiseptic treatments and according to present knowledge, allergic or toxic reactions are not expected [4].

At present, several different approaches are followed to produce CAP, e.g., the plasma jet and the floating electrode dielectric barrier discharge. The disadvantage of the usage of the plasma jet for wound treatment is the inhomogeneous plasma distribution and, in the case of the floating electrode, the small distance between the electrode and the skin (2 mm). By using a plasma torch these problems can be overcome: The plasma itself is produced by six electrodes inside of the plasma torch and subsequently delivered to the skin by an argon gas flow which ensures a homogeneous distribution of the plasma species on the treated skin. This plasma device, called MicroPlaSter®, was developed in close cooperation with the company ADTEC Plasma Technology Co. Ltd, Hiroshima, and was especially designed for homogenous wound treatment [5].

First clinical trials using the MicroPlaSter α® with a daily treatment time of 5 min showed the efficacy and tolerability of CAP for infected chronic wounds [3]. In a follow-up study the next generation device (MicroPlaSter ß®) clearly demonstrated that 2 min of plasma were sufficient to achieve a highly significant reduction of the bacterial load on chronic infected wounds in patients [2]. Wound healing was also improved and demonstrated in a first placebo-controlled study on acute skin graft donor sites using the MicroPlaSter ß® [1]. In comparison to the placebo treatment, a daily 2 min plasma treatment led to faster re-epithelialisation, less fibrin layers and blood crusts without side effects or negative influences on the wound surroundings [1].

Nevertheless there are still many open issues with regard to the mechanisms of action of plasma on mammalian cells and tissues in wound repair, both from the biological and the physical perspective.

A major future task will be the introduction of CAP into daily clinical medicine. Therefore, it is important to investigate the molecular changes and mechanisms which are responsible for the observed effects.

## Materials and Methods

### Plasma Device and Treatment of Cells

The CAP device employed in this study was the MicroPlaSter ß® plasma torch system (Microwave 2.45 GHz, 110 W, argon flow 4.0 l/min, treatment diameter ∼5 cm) developed and built by the Max Planck Institute for Extraterrestrial Physics in Garching/Germany and the company ADTEC Plasma Technology Co. Ltd., Hiroshima/London. The same device was also used in clinical studies for the treatment of chronic and acute wounds [1]–[3]. The six electrode plasma torch (**figure S1a**) of the MicroPlaSter ß® (**figure S1b**) produces atmospheric plasma at a few degrees above room temperature thereby generating a mix of active components such as charged particles (electrons, ions), reactive oxygen (ROS) and nitrogen species (RNS) and UV photons [2], [5]. These components are delivered to the respective target (e.g. the skin) by an applied argon gas flow. The measured UV power was 60 µW/cm^2^. The optical emission spectrum showed emission in the UVA, UVB, and UVC range. Nevertheless most of the UV emission appeared in the UVB range, so that only minimal UVC emission was detected. The optical emission spectrum of the plasma discharge moreover showed that NO is present in the plasma system as the lines in the UVC region correspond to NO molecules (NO γ system) (**figure S1c**).

For the CAP treatment 50.000 cells were seeded in 35 mm Petri dishes (Greiner bio one; Frickenhausen, Germany). Immediately before CAP exposure the medium DMEM was removed and the cells were covered with 750 µl PBS. This liquid film is necessary to protect the cells against dehydration during exposure. The plasma torch was placed at a distance of 20 mm from the Petri dish. At this distance the temperature does not exceed 37°C. The cells were exposed to CAP for 2 min in analogy to the treatment time for wounds [2]. Immediately after the CAP treatment PBS was replaced by 2.5 ml DMEM. Control cells were handled in the same manner except for the exposure to CAP.

### Cell Culture Conditions

Primary human dermal fibroblasts (2F0621, 9F0438, 9F0889) were purchased from Lonza (Verviers, Belgium) and were cultured according to [6].

### Human Apoptosis Array

Human Apoptosis Arrays (array kit ARY009, R&D systems, Minneapolis, USA) were performed according to the manufactureŕs instructions and allow a parallel determination of 35 apoptosis related proteins. Cellular extracts were collected from fibroblasts 24 h after 2 min of CAP treatment or from untreated control cells. For isolation of cellular extracts fibroblasts were lysed in 200 µl RIPA-buffer (Roche Applied Science, Mannheim, Germany) and incubated for 15 min at 4°C. Insoluble fragments were removed by centrifugation at 13 000 r.p.m. for 10 min and the supernatant lysate was immediately shock frozen and stored at −80°C. Arrays were performed in duplicates with three different fibroblast cultures. Densitometry of the array spots was performed using MethaMorph-Software (Molecular Devices, Summyvale, USA).

### Human Cytokine Array

Human Cytokine Arrays (array kit ARY005, R&D systems, Minneapolis, USA) were performed using cell supernatants, collected from fibroblasts 24 h after a 2 min CAP treatment or from untreated control cells. The arrays were conducted according to the manufactureŕs instruction and offer a parallel determination of 36 cytokines. Arrays were performed in duplicates with three different fibroblast cultures. Densitometry of the array spots was performed in analogy to the Human Apoptosis Array.

### FlowCytomix™ Simplex Technology

The Human FlowCytomix™ Simplex Detection System (eBioscience, San Diego, CA 92121, USA) was used to quantify the protein amounts of IL-1 beta, IL-1 alpha, IL-6, IL-8, IL-10, IL-23, IL-27, IFN-gamma, TNF-alpha, MCP-1, MIP-1 beta, MIP-1 alpha, G-CSF, MIG, MMP-9, TIMP-1, Caspase-3 and Caspase-9 from cell supernatants collected 24 h, 48 h, and 72 h after a 2 min CAP treatment or from untreated control fibroblasts according to the manufactureŕs instruction. The concentrations of IFN-alpha, IFN-gamma, TNF-alpha, IL-1 alpha, IL-10, G-CSF, MIG and MMP-9 were below detectable levels. Each sample was assayed in duplicates, and the entire experiments were performed three times with three different fibroblast cultures.

### Enzyme-linked Immunosorbent Assay (ELISA)

Cell supernatants were collected 24 h, 48 h, and 72 h after a 2 min CAP treatment or of untreated control fibroblasts and were analyzed by ELISAs, respectively, according to the manufacturer’s instructions. ELISAs for detection of TGF-ß1 and TGF-ß2 were received from R&D Systems, Wiesbaden-Nordenstadt, Germany. Each sample was assayed in duplicates, and the entire experiments were performed three times with three different fibroblast cultures.

### Analysis of mRNA Expression by Quantitative RT-PCR

Total cellular RNA was isolated from fibroblasts 6 h, 24 h, 48 h, and 72 h after the CAP treatment using the RNeasy kit (Marchery-Nagel, Düren Germany) and cDNAs were generated by reverse transcriptase reaction according to [7]. Quantitative RT-PCR was performed with specific sets of primers and conditions (table S1) applying LightCycler technology (Roche Diagnostics, Mannheim, Germany) as described [7]. Each RT-PCR was performed in duplicates with cDNA of at least three different fibroblast cell cultures.

### Proliferation Assay (XTT)

24 h, 48 h and 72 h after CAP exposure for 2 min the cells were removed from the Petri dishes and cell proliferation was determined using the XTT Proliferation Assay (Roche Diagnostics, Mannheim, Germany) according to [8]. Each sample was assayed in duplicates, and the entire experiments were performed three times with two different fibroblast cell lines.

### Wound Healing Assay (Scratch Assay)

The migratory behaviour of fibroblasts was assayed by means of a wound healing assay using a culture-insert (ibidi GmbH, Am Klopferspitz 19, Martinsried, Germany) according to the manufacturer’s instruction. After 24 h the culture-insert was removed leaving a cell-free gap (“defined wound”) of approx. 500 µm. The migration rate into this “wound area” was documented and measured after 12 h and 22 h using a Carl Zeiss microscope (Carl Zeiss Vision GmbH, Halbergmoos, Germany). The migration rate of untreated fibroblasts was set to 100% and was compared with CAP treated cells for 30 sec. A reduced CAP treatment time was required for this assay because 2 min of CAP treatment results in cell-detachment of the confluent cell layer. Each analysis was performed in duplicates and was repeated two times for two different fibroblast cultures.

### Mouse Experiments

129Sv/Ev wild-type mice were obtained from the Robertson lab (Department of Molecular & Cellular Biology, Harvard University, Cambridge, MA02138). All animal experiments were conducted with appropriate permission from the animal rights commission of the state of Bavaria and maintained in agreement with the European Union guidelines. All experiments were approved by the Committee on the Ethics of Animal Experiments of the University of Regensburg, Germany (Permit Number: 54-2532.1-10/11). All surgery was performed under anesthesia, and all efforts were made to minimize suffering.

The animals were 8–12 weeks of age at the beginning of the experiment. The mice were anesthetized using Ketamine (100 mg/kg body weight) and Xylazine (5 mg/kg body weight), animalś backs were shaved and two 6 mm full-thickness wounds per mouse were generated using a 6 mm dermal biopsy punch (Stiefel, GSK company, Germany) on both sides of the dorsal midline on 8 mice per experiment. The wounds were allowed to dry to form a scab. 4 mice became a daily CAP treatment using the MicroPlaSter ß® (110 W, argon flow 4 l/min) for 2 min, 10 days long. Control mice were treated with the placebo modus of the MicroPlaSter ß® for 2 min instead of plasma, to resemble the same stress-situation for all animals. All wounds and wound margins as well as mouse behaviour were observed carefully to exclude side effects of the CAP or placebo treatment. Mice were sacrificed on day 5 and day 15 after wounding and an area of 7 mm in diameter which included the complete epithelial margins were excised. Paraffin sections from the wound tissue were stained with hematoxylin/eosin, Sirius Red/Fast Green and immunohistology was performed directed against CD68 (Dako, Carpinteria, CA) according to [9], anti-alpha-smooth muscle actin (alpha-SMA) (Dako) according to [10], and Ly6G (clone 1A8, BD Biosciences, California, USA; 1/50). Evaluation of the staining was performed semi-quantitatively by means of light microscopy (Carl Zeiss Vision, Hallbergmoos, Germany) in magnification as indicated.

For mRNA expression analysis wound tissue was cut and dermis was separated from epidermis by dispase digest (Life Technologies GmbH, Darmstadt, Germany) over night at 4°C. Total dermal RNA was subsequently isolated and complementary DNA was generated as described elsewhere [6]. mRNA expression was analyzed by quantitative RT-PCR as described above.

### Statistical Analysis

Results are expressed as the mean ± s.d. (range) or a percentage value. Comparisons between groups were made using Student’s unpaired t-test. A p value <0.05 was considered statistically significant (*p<0.05; **p<0.01; ***p<0.001; ns: not significant). All calculations were performed using the GraphPad Prism software package (GraphPad Software Inc., San Diego, CA, USA).

## Results

The aim of this study was to evaluate the influence of CAP using the MicroPlaSter ß® technology on human dermal fibroblasts to determine molecular changes of wound healing relevant genes and functional effects after a single CAP treatment of 2 min. Mouse wound healing experiments were supplemented to confirm our *in vitro* findings.

### Increased Production of Cytokines and Growth Factors in Fibroblasts after CAP Treatment

The repair process after wounding is initiated immediately after injury by the release of various growth factors and cytokines [11]. Cytokines and growth factors are important key molecules during wound healing and we were interested whether CAP influences those factors.

Fibroblasts were exposed to CAP for 2 min or remained untreated and were further incubated for 24 h before supernatants were collected and processed for determination of protein amounts using a cytokine array. Interestingly, we observed significant higher amounts of several proteins e.g. CD 40 Ligand (CD154), GRO alpha (CXCL1), IL-1 ra (IL-1F3), IL-6, IL-8, MCP-1 (CCL2), and Serpine E1 (PAI-1) (**table 1** **I.**) in the supernatants of CAP treated fibroblasts. These results suggest that activation of those factors is an important mechanism triggered by the CAP treatment.

**Table 1:** Protein Array results.

Name	Fold change (CAP/control)	P value
**I. Cytokine Array**		
CD40 Ligand (CD154)	1.266+/−0.0109	0.0004 (***)
GRO alpha (CXCL1)	3.295+/−0.5068	0.0118 (*)
IL-1 ra (IL-1F3)	2.894+/−0.0692	0.0003 (***)
IL-6	1.900+/−0.1045	0.0028 (**)
IL-8	1.810+/−0.0284	0.0003 (***)
MCP-1 (CCL2)	11.213+/−1.8975	0.0011 (**)
Serpine E1 (PAI-1)	2.284+/−0.0068	0.0001 (***)
**II. Apoptosis Array**		
**Pro-apoptotic:**		
Bad	0.436+/−0.1660	0.0065 (**)
Bax	0.598+/−0.2548	0.0513 (ns)
Bcl-x	0.615+/−0.2193	0.0394 (*)
Cleaved-Caspase-3	0.572+/−0.1424	0.0092 (**)
Cytochrome c	0.874+/−0.4492	0.613 (ns)
Phospho-p53 (S15)	0.640+/−0.2905	0.1183 (ns)
SMAC/Diablo	0.662+/−0.3320	0.1344 (ns)
**Anti-apoptotic:**		
Bcl-2	0.634+/−0.2896	.0394 (*)
cIAP-1	0.588+/−0.2679	0.0542 (ns)
HSP70	0.901+/−0.2600	0.5028 (ns)
Survivin	0.534+/−0.1704	0.0120 (*)

Fold change (CAP/control) and p value of selected molecules from I. Cytokine Array and II. Apoptosis Array.

* p<0.05;

** p<0.01;

*** p<0.001;

ns: not significant.

**Figure 1:**
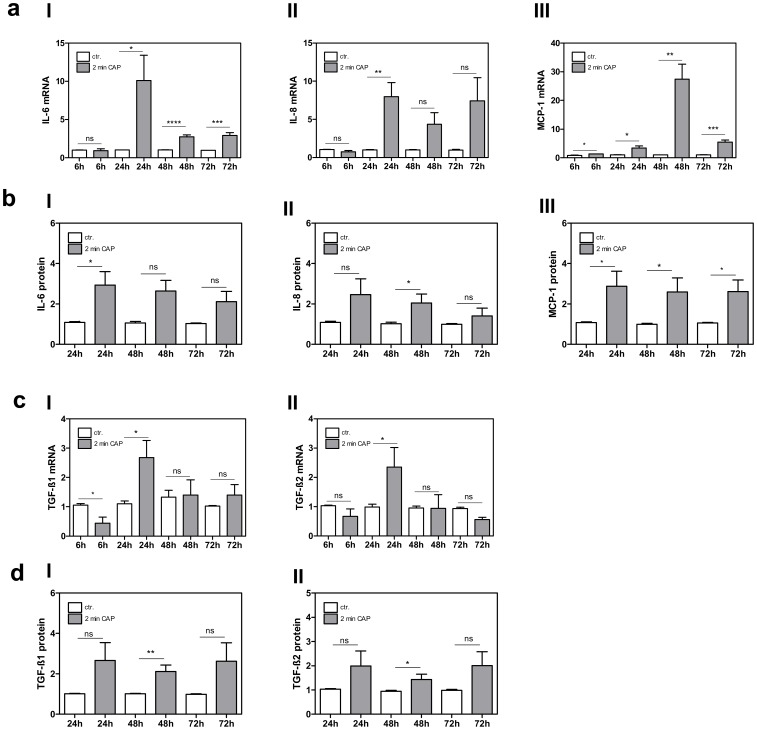
Expression of Cytokines and Growth Factors after CAP treatment. (**a I–III**) mRNA expression analysis of IL-6, IL-8 and MCP-1 performed 6 h, 24 h, 48 h and 72 h after CAP treatment for 2 min by LightCycler® 1.2 technology. (**b I–III**) Protein expression analysis of IL-6, IL-8 and MCP-1 24 h, 48 h and 72 h after CAP treatment for 2 min by FlowCytomix™ Technology. (**c I–II** and **d I–II**) Expression profile of TGF-ß1 and TGF-ß2 on mRNA level analyzed by LightCycler® 1.2 technology and on protein level analyzed by ELISA technique is shown in accordance to the above mentioned time points after CAP treatment for 2 min. *p<0.05; **p<0.01; ***p<0.001; ns: not significant.

Array results were confirmed exemplarily and analysis was performed 6 h, 24 h, 48 h and 72 h after CAP treatment for IL-6, IL-8 and MCP-1 on mRNA level (**figure 1a** **, I–III**) and after 24 h, 48 h and 72 h on protein level by FlowCytomix™ Technology (**figure 1b** **, I–III**).

We were further interested in the expression profile of TGF-ß1 and TGF-ß2, the most studied growth factors in the wound healing scenario [11], [12], not covered within the cytokine array. TGF-ßs regulate many important events during wound healing including cell migration and proliferation, synthesis of extracellular matrix, angiogenesis, remodelling and increase the rate of healing and the breaking strength of the repaired tissue [13].

We observed that both molecules are significantly induced 24 h after CAP treatment on mRNA level **(** **figure 1c** **, I–II**), and 48 h after exposure on protein level analyzed by ELISA technique (**figure 1d** **, I–II**), suggesting that CAP triggers this wound healing-important growth factors in fibroblasts.

### The Apoptotic Machinery was not Affected by CAP Treatment in Fibroblasts

It is described in several studies that CAP induces necrosis or apoptosis in tumour cells [14]–[17]. Only a few *in vitro* studies are available analyzing CAP effects on normal cells. With regard to wound healing it is absolutely important to know whether CAP damages normal cells and induces the apoptotic machinery. We used a Human Apoptosis Array which detects 35 apoptosis-related proteins and searched for candidates differentially expressed 24 h after a 2 min CAP treatment of fibroblasts. The evaluation shows that no pro-apoptotic or anti-apoptotic factors were significantly upregulated (table 1 II.), suggesting that the apoptotic machinery was not affected by CAP in fibroblasts.

### Induction of Fibroblast Migration without Changing Cell Proliferation after CAP Treatment

A further important step during wound healing is the proliferation of fibroblasts and their migration into the wound area [18].

**Figure 2:**
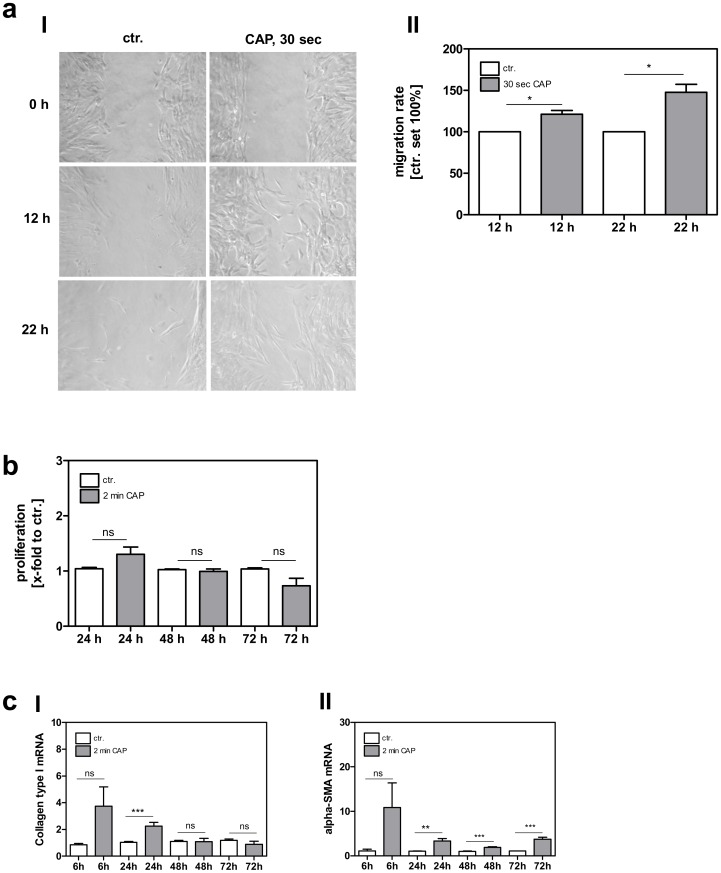
Fibroblast migration, proliferation and ECM production after CAP treatment. (a I) A wound healing assay shows that CAP treatment for 30 sec promotes fibroblast migration. Representative images are shown immediately after culture insert was removed (0 h) and 12 h and 22 h later. (**a II**) The migration rate was calculated 12 h and 22 h after culture insert was removed and is displayed as the percentage relative to untreated control (ctr. set 100%). The results are measurements of the “wound area” from at least four separate visual fields from three separate experiments. (**b**) Cell proliferation was determined using XTT proliferation assay 24 h, 48 h and 72 h after CAP exposure for 2 min. (**c I–II**) mRNA expression analysis of collagen type I and alpha-SMA was determined 6 h, 24 h, 48 h and 72 h after CAP treatment for 2 min by LightCycler® 1.2 technology. *p<0.05; **p<0.01; ***p<0.001; ns: not significant.

A wound healing assay was performed and an accelerated migration rate of CAP treated fibroblast was observed after 12 h and 22 h (**figure 2a** **I–II**).

Interestingly, proliferation of fibroblasts was not significantly influenced 24 h, 48 h, and 72 h after CAP treatment for 2 min (**figure 2b**).

These data support a positive effect of CAP on fibroblast migration, whereas fibroblast proliferation remained unchanged after CAP treatment.

### Accelerated Collagen Synthesis and alpha-SMA Expression after CAP Treatment

Important during wound healing is the production of ECM components. Hereby, activated fibroblasts (alpha-SMA-expressing myofibroblasts) are the cells responsible for ECM production and remodelling during wound healing. We observed that collagen type I and alpha-SMA mRNA expression are both induced after CAP treatment (**figure 2c** **I–II**), suggesting that CAP influences collagen synthesis and activation of fibroblasts.

### Improved Wound Healing after CAP Treatment in Mice

**Figure 3:**
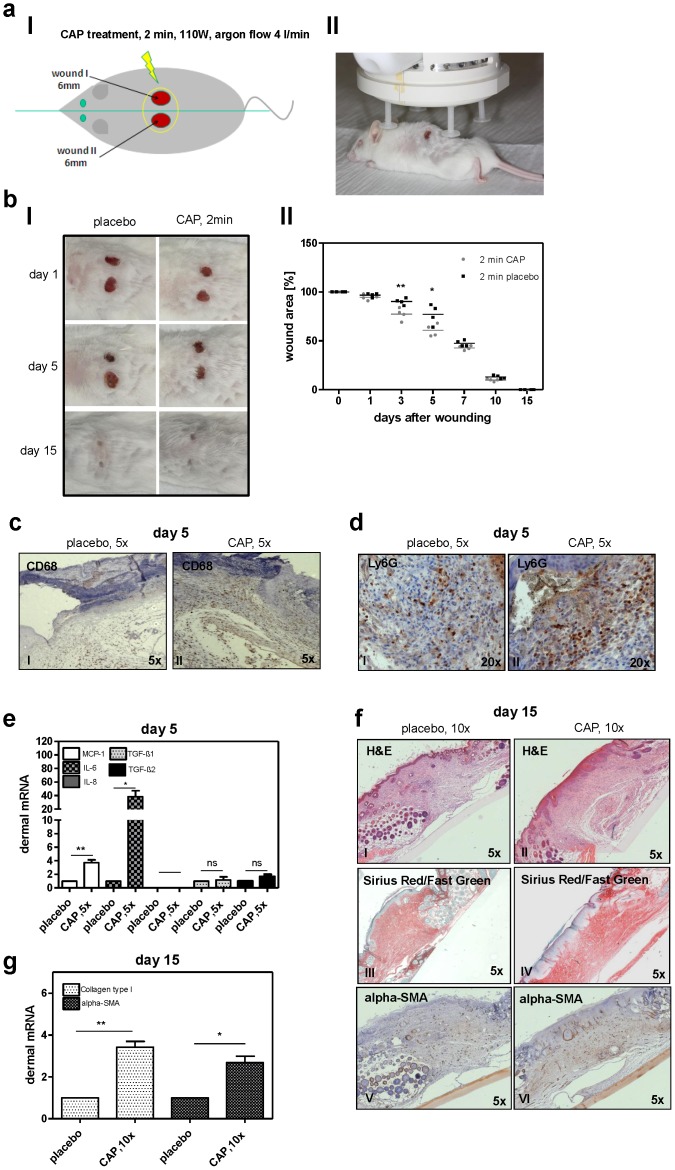
Wound healing after CAP therapy in a mouse wound healing model. (**a I**) Schematic overview of the wound generation in 129/Sv/Ev mice. (**a II**) CAP treatment of wounds with the MicroPlaSter ß® device (2 min daily); control mice were placebo-treated. (**b I**) Representative photographs of placebo or CAP treated wounds at the indicated days after wounding. (**b II**) Quantification of the wound area at the indicating times after wounding. Results represent the mean+/− s.e.m; n = 4 for each time point. *p<0.05; **p<0.01. Representative examples of (**c**) CD68 and (**d**) Ly6G immunohistological staining of skin wounds 5 days after wounding with 5 × placebo or 5 × CAP therapy. The amount of neutrophils was determined by counting the positive stained cells per 4 different fields of view in magnification as indicated (**e**) mRNA expression of MCP-1, IL-6, IL-8, TGF-ß1 and TGF-ß2 in wound tissue on day 5 after wounding after 5 × placebo or 5 × CAP therapy. *p<0.05; **p<0.01; ns: not significant; nd: not detectable. Representative examples of H&E (**f I–II**), Sirius Red/Fast Green (**f III–IV**) and alpha-SMA (**f V–VI**) staining of skin wounds 15 days after wounding with 10 × placebo or with 10 × CAP therapy. Magnification as indicated. (**g**) Dermal mRNA expression of collagen type I and alpha-SMA 15 days after wounding with 10 × CAP therapy compared to placebo control. *p<0.05; **p<0.01.

Next to the *in vitro* analysis of fibroblasts, we were interested in the complex process *in vivo* using a mouse wound healing model. Two 6 mm full thickness wounds were generated on both sides of the dorsal midline (**figure 3a** **I**) and mice became a daily CAP therapy for 2 min using the MicroPlaSter ß® (**figure 3a** **II**). Control mice were treated with the placebo modus of the MicroPlaSter ß® (argon gas without plasma). The wound area was measured daily and CAP treated mice showed a significantly improved wound closure on day 3 and on day 5. Both wounds, from placebo and CAP treated mice, were completely closed after 15 days (**figure 3b** **I–II**). During this time mice showed normal behaviour and nutrition and wounds as well as wound surroundings were completely inconspicuous in regard to possible side effects of treatment (e.g. wound infection, erythema, swelling, oozing). Interestingly, obvious differences in favour of CAP treatment were observed histologically. On day 5 after wounding, we detected an increased CD68 staining in skin sections after 5 × CAP therapy compared to placebo treatment (**figure 3c**), suggesting an increased amount of macrophages due to an elevated or accelerated immune defence in the early phases of wound healing where macrophages play an important role to clean the wound area by phagocytosis and secrete growth factors that further activate wound healing. Additionally, we observed a 30% induction of neutrophils in the wound area on day 5 after wounding after CAP therapy compared to the placebo control (**figure 3d**), whereas no significant difference in the amount of neutrophils was observed on day 15 after wounding (data not shown). These observations suggest that CAP accelerates neutrophil immigration in the early phases of wound healing without protracted increase in later phases.

Next, we analyzed the expression level of these molecules, which were significantly affected by CAP in our *in vitro* study. Interestingly, we observed that MCP-1 and IL-6 were also induced in our *in vivo* approach on day 5 after wounding, whereas TGF-ß1 and TGF-ß do not differ between the CAP and the placebo treatment. IL-8 expression was detectable neither in placebo nor in CAP treated wounds on day 5 after wounding (**figure 3e**). Furthermore, at day 15 after wounding, we observed a thicker epidermal layer after the CAP treatment by H&E staining (**figure 3f** **I–II**), suggesting a positive progress of re-epithelialization. Sirius Red/Fast Green staining showed tightly arranged collagen fibers after the CAP treatment compared to a loose connective tissue after the placebo treatment (**figure 3f** **III–IV**) and alpha-SMA staining revealed more positivity after CAP therapy on skin sections generated 15 days after wounding (**figure 3f** **V–VI**). Assessment of collagen type I and alpha-SMA dermal mRNA expression of wound tissues affirmed a significantly higher expression of both molecules on day 15 after a 10 × CAP therapy (**figure 3g**).

In summary, our *in vivo* results approved our *in vitro* findings and give evidence that CAP affects wound healing by inducing important wound healing relevant molecules.

## Discussion

Skin-wound healing is an orchestrated biological process consisting of three sequential phases: an inflammatory, a proliferative, and a remodelling phase.

During the inflammatory phase fibroblasts are involved in the secretion of cytokines and growth factors to activate the immune defence [19]. During the proliferative- and remodelling phase fibroblasts are important for tissue granulation and reorganization of the provisional extracellular matrix (ECM) [18].

In the present study we focused on the expression profile of wound healing-relevant molecules and functional changes of fibroblasts after CAP treatment. To implicate the cellular interplay, we added *in vivo* wound healing experiments to complement our *in vitro* findings.

Analyzing the expression status of cytokines and growth factors in fibroblasts after CAP treatment, we observed a significant induction of several pro-inflammatory factors, like IL-6, IL-8, MCP-1 or TGF-ß1/2, which are known to be expressed immediately after cutaneous injury by fibroblasts, keratinocytes and macrophages to recruit and activate inflammatory cells [12], [20], [21]. Also the chemokine GRO alpha (CXCL-1) which has neutrophil chemo-attractant activity and is involved in inflammation and wound healing [22], [23] was significantly induced by CAP treatment. An induction was also shown for Serpine E1 (PAI-1) which inhibits proteases and regulates tissue remodelling [24] and CD154 (CD40 ligand) which promotes recruitment of leukocytes to lesions and leads to chemokine and cytokine production [25].

We suggest that an over-expression and secretion of above mentioned molecules by fibroblasts after CAP treatment might be responsible for a better and accelerated immune defence during the inflammatory phase.

This suggestion is in line with our *in vivo* findings in the early phase of wound healing (5 days after wounding) showing increased accumulation of inflammatory macrophages and neutrophils after the CAP treatment. These results are in accordance with the study of Yu and colleagues, showing that acute inflammation is promoted by the plasma treatment and peaked on day 4 after wounding, earlier than in the control group in which acute inflammation reached a peak on day 7 after wounding [26].

Interestingly, the highest difference in expression using protein array technology was obtained for MCP-1 (11-fold induction compared to control), whereas the induction was only about 3-fold in the FlowCytomix experiment 24 h after CAP treatment. These data obtained using different methods suggest that MCP-1 is an important factor affected by CAP treatment. MCP-1 is a potent chemokine which is able to stimulate collagen type I expression in skin fibroblasts indirectly via endogenous up-regulation of TGF-ß expression [27]. In our *in vitro* study, we observed an accelerated expression of collagen type I, alpha-SMA, TGF-ß1 and TGF-ß2 after CAP treatment in fibroblasts, suggesting that CAP promotes expression of a cascade of essential molecules important for wound healing. Within our *in vivo* wound healing model we could confirm an induction of MCP-1 and IL-6, whereas a significant induction of TGF-ß1 and TGF-ß2 was not observed on day 5 after wounding. We suggest that MCP-1 and IL-6 are molecules, which are affected “early” by CAP, and TGF-ß1 and TGF-ß2 are affected “later” so that differences in expression of those molecules are not visible on day 5 after wounding.

Evidence that CAP is involved in different cellular mechanisms was further obtained by functional assays. Interestingly, we observed that CAP influences fibroblast migration analyzed in a scratch wound healing assay without having an effect on the cell proliferation analyzed by XTT method. Up to now, we do not know how CAP influences these mechanisms leading to above mentioned findings but with regard to wound healing therapy it seems to be beneficial that 2 min of CAP has no influence on the fibroblast proliferation. Hence, an excessive fibroblast proliferation is in accordance with excess production of ECM. A potential overgrowth of scar by excessive growth and invasion of fibroblasts beyond the original injury, resulting in keloid formation, is thus not expectable by CAP therapy using the MicroPlaSter ß®.

Kalghatgi and co-workers revealed that the application of CAP created by Floating Electrode Dielectric Barrier Discharge (FE-DBD) for short exposure times down to 30 s induces proliferation of endothelial cells while the treatment with longer exposure times (>60 s) induces apoptosis [28]. In another study by Tipa and Kroesen the authors used a scratch wound healing assay with murine 3T3 fibroblasts and reported that the effect of CAP (for the treatment, a cold atmospheric plasma needle (13.56-MHz microjet in helium) was used) results in increased proliferation [29]. In our study we adapted the CAP treatment-time to the current clinical standard (2 min) using the MicroPlaSter ß®. UV radiation generated by argon plasma limits the duration of wound treatment to 2 min/treatment, because 7.6 mJ/cm^2^, which is calculated without the ICNIRP weighting function (equivalent to 4 min of irradiation with the MicroPlaSter®) is the pro-apoptotic limit of total UV radiation produced by argon plasma and temporary inhibits fibroblast proliferation.

In addition, we want to point out that each CAP device has its own characteristics, i.e. the variation of active plasma components strongly depends on the plasma production mechanisms (electrode configuration, resource gas, etc.). For instance, electrical current passes through the tissue if FE-DBD is used. This is not the case for the MicroPlaSter ß®, which produces CAP indirectly. Moreover, the amount of reactive species produced by FE-DBD is higher than for our device. FE-DBD uses the surrounding air for plasma production which leads to high amounts of reactive oxygen and nitrogen species. For the MicroPlaSter ß® the reactive species are produced by mixing of the argon plasma with the ambient air. Therefore only “low” amounts of reactive oxygen and nitrogen species are produced.

The MicroPlaSter® was specifically “designed” for disinfection of large chronic wounds [30] – i.e. for inactivating bacteria without harming eukaryotic cells. Amongst all components produced by the plasma torch the short-wavelength UV radiation (UVC) has presumably the most damaging effect on human skin cell viability and proliferation. The optical spectrum of the argon plasma-generated UV (**figure S1c**) shows that the UVC intensity is very low, suggesting that a 2 min plasma treatment with the MicroPlaSter® is not harmful for eukaryotic cells. The clinical study by Isbary et al. further showed the efficiency of this plasma composition with regard to bacterial reduction on chronic wounds [2]. Moreover, as already mentioned earlier direct positive effects on wound healing were observed in acute skin graft donor sites without side effects [1]. These results are in line with the clinical data from Metelmann and colleagues, who also were able to show a healing benefit by CAP in the treatment of laser induced artificial skin wounds [31].

Our *in vitro* human and *in vivo* animal data show that CAP is able to trigger key events on a cellular level which are important for wound healing (e.g. migration of fibroblasts, activation of immune deficiencies, regulation of collagen synthesis).

It is known from the literature that many cells participate in NO synthesis during the proliferative phase of wounding. Endogenous NO release regulates collagen formation and wound contraction during wound healing [32]. The exact mechanisms of NO action on wound healing parameters are still unknown but we suggest that the exogenic NO generated by the plasma torch of the MicroPlaSter ß® positively affects above mentioned key events of wound healing and might be responsible for the observed cellular effects.

In summary, our results reveal that a 2 min CAP treatment using the MicroPlaSter ß® technology improves wound healing in vitro and in vivo by inducing different wound healing relevant molecules without causing apoptosis in human fibroblasts. Future studies must elucidate the connection between CAP and the affected molecules to become an idea on the mechanism of CAP on eukaryotic cells.

## Supporting Information

Figure S1 **Plasma torch, plasma device and optical emission spectrum of the plasma discharge.** (**a**) The plasma torch consists of 6 stainless steel electrodes. The centers of the 6 electrodes are distributed equally at a distance of 6 mm from the inner surface of the cylinder. 6 small plasmas are produced between each of the electrode’s tips and the inner surface of the cylinder. (**b**) MicroPlaSter ß® version of the atmospheric plasma device for wound treatment (Max-Planck Institute for Extraterrestrial Physics). (**c**) Optical emission spectrum of the plasma discharge shows that argon plasma produces polychromatic UV radiation with two main peaks in the UVB and UVA ranges. The optical emission spectrum was measured by a spectrometer. This data gives the information of relative intensity at different wavelength. Additionally, the UV power was measured at the position of the plasma treatment. This is an absolute power at the position. With the spectrum and the measured power, the power spectrum at different wavelength can be obtained. Using the spectral weighting function provided in ICNIRP guidelines, the effective power spectrum is calculated. At the end, by summing the power spectrum up with the wavelength, the effective power is obtained.
(TIF)

Table S1 **Primers and conditions.**
(DOCX)
