# Supplementary figures and images for: Cold Atmospheric Plasma (CAP) Changes Gene Expression of Key Molecules of the Wound Healing Machinery and Improves Wound Healing In Vitro and In Vivo
Source: PLoS One. 2013 Nov 12;8(11):e79325. doi: 10.1371/journal.pone.0079325 (PMC3825691; doi:10.1371/journal.pone.0079325)

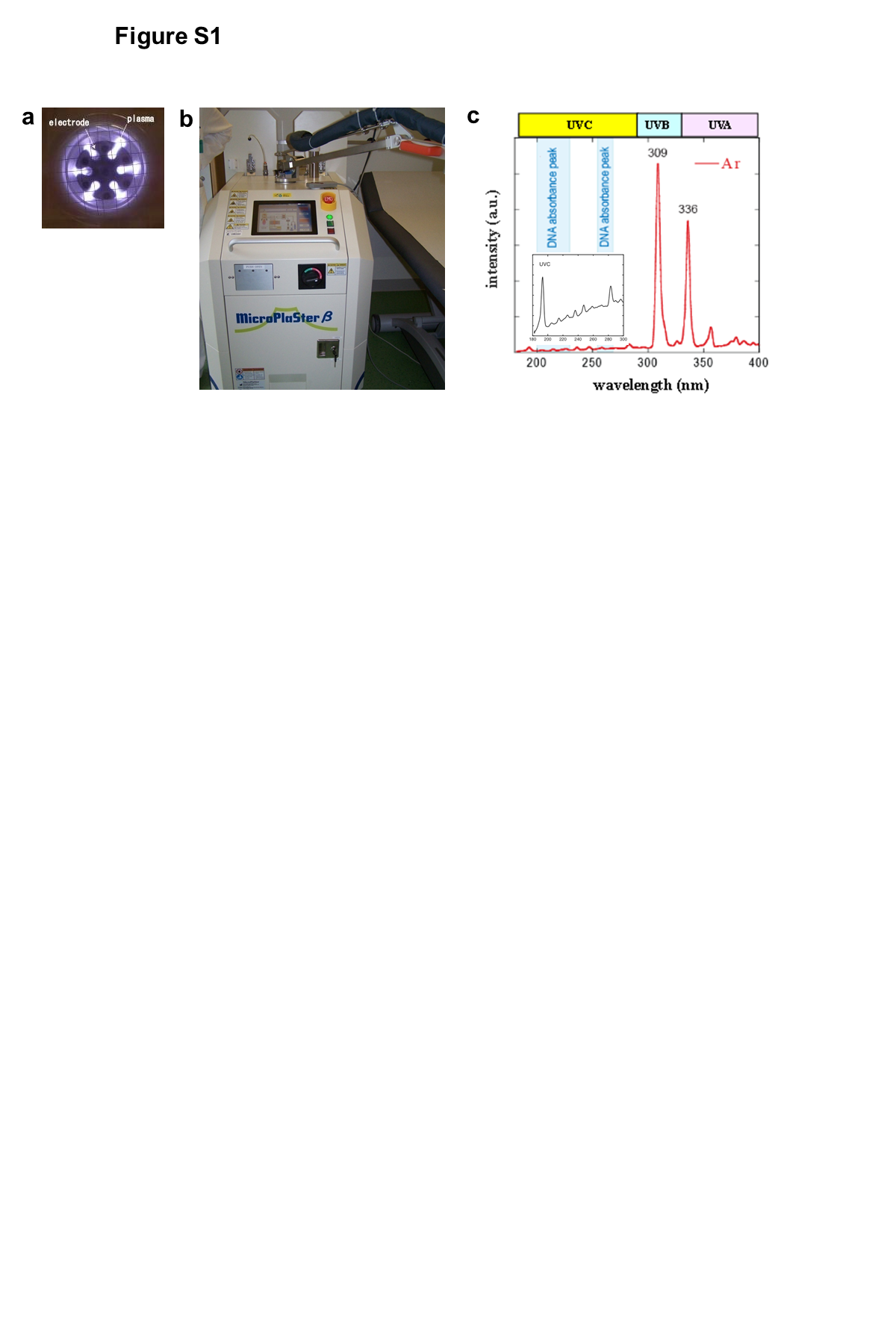

Supplement: Figure S1 — Plasma torch, plasma device and optical emission spectrum of the plasma discharge. (a) The plasma torch consists of 6 stainless steel electrodes. The centers of the 6 electrodes are distributed equally at a distance of 6 mm from the inner surface of the cylinder. 6 small plasmas are produced between each of the electrode’s tips and the inner surface of the cylinder. (b) MicroPlaSter ß® version of the atmospheric plasma device for wound treatment (Max-Planck Institute for Extraterrestrial Physics). (c) Optical emission spectrum of the plasma discharge shows that argon plasma produces polychromatic UV radiation with two main peaks in the UVB and UVA ranges. The optical emission spectrum was measured by a spectrometer. This data gives the information of relative intensity at different wavelength. Additionally, the UV power was measured at the position of the plasma treatment. This is an absolute power at the position. With the spectrum and the measured power, the power spectrum at different wavelength can be obtained. Using the spectral weighting function provided in ICNIRP guidelines, the effective power spectrum is calculated. At the end, by summing the power spectrum up with the wavelength, the effective power is obtained. (TIF) [file pone.0079325.s001.tif]
