# Supplementary material for: Cold Atmospheric Plasma (CAP) Changes Gene Expression of Key Molecules of the Wound Healing Machinery and Improves Wound Healing In Vitro and In Vivo
Source: PLoS One. 2013 Nov 12;8(11):e79325. doi: 10.1371/journal.pone.0079325 (PMC3825691; doi:10.1371/journal.pone.0079325)
Supplement: Table S1 — Primers and conditions. (DOCX) [file pone.0079325.s002.docx]

| ***Primer name*** | ***Forward primer*** | ***Reverse primer*** | ***Condition***  ***(annealing, melting)*** |
| --- | --- | --- | --- |
| _human collagen I α1_ | _5’-CGGCTCCTGCTCCTCTT-3’_ | _5’-GGGGCAGTTCTTGGTCTC-3’_ | _ann. 68 °C, mt 82 °C_ |
| _human TGF-ß1_ | _5'-GCAGAGCTGCGTCTGCTGAGGC-3'_ | _5'-CCCGTTGATGTCCACTTGCAGTG-3'_ | _ann. 65 °C, mt 85 °C_ |
| _human TGF-ß2_ | _5'-TCTAGGGTGGAAATGGATACACGAACC-3'_ | _5'-TGTTACAAGCATCATCGTTGTCGTCG-3'_ | _ann. 65 °C, mt 78 °C_ |
| _human alpha-SMA_ | _5’-GGCCGAGATCTCACTGACTAC-3’_ | _5’-TTCATGGATGCCAGCAGA-3’_ | _ann. 58 °C, mt 84 °C_ |
| _human MCP-1_ | _5'-CGCGAGCTATAGAAGAATCAC-3'_ | _5'-TTGGGTTGTGGAGTGAGTGT-3'_ | _ann. 68 °C, mt 82 °C_ |
| _human ß-actin_ | _5'-TACGTCGCCCTGGACTTCGAGC-3'_ | _5'-GATGGAGCCGCCGATCCACACGG-3'_ | _ann. 62 °C, mt 78 °C_ |
| _human IL-8_ | _5'-CTGCAGCTCTGTGTGAAGGTG-3'_ | _5'-ACAGAGCTCTCTTCCATCAG-3'_ | _ann. 60 °C, mt 81 °C_ |
| _human IL-6_ | _5'-TGCAATAACCACCCCTGACC-3'_ | _5'-GTGCCCATGCTACATTTGCC-3'_ | _ann. 60 °C, mt 84 °C_ |
| _murine collagen I α1_ | _5´-CAACGAGATCGAGCTCAGA-3´_ | _5´-CTAGTCCGAATTCCTGGTCT-3´_ | _ann. 60 °C, mt 86 °C_ |
| _murine alpha-SMA_ | _5´-GGAGTAATGGTTGGAATGGGC-3´_ | _5´-AGGGTTCAGTGGTGCCTCTG-3´_ | _ann. 60 °C, mt 84 °C_ |
| _murine IL-6_ | _5´-TTCACAAGTCCGGAGAGGAG-3´_ | _5´-AGGAGAGCATTGGAAATTGG-3´_ | _ann. 60 °C, mt 84 °C_ |
| _murine TGF-ß1_ | _5´-GGCTCTGGAGAACAGCACATC-3´_ | _5´-CAAGCAGTCCTTCCCTTCAGG-3´_ | _ann. 60 °C, mt 89 °C_ |
| _murine TGF-ß2_ | _5´-TGGCGCTCAGTCTGTCTACCT-3´_ | _5´-TTGGCGTAGTACTCCTCGTCG-3´_ | _ann. 60 °C, mt 89 °C_ |
| _murine IL-8_ | _5´-CTAGGCATCTTCGTCCGTCC-3´_ | _5´-GAGGTCTCCCGAATTGGAAAG-3´_ | _ann. 60 °C, mt 83 °C_ |
| _murine MCP-1_ | _5´-TGGGCCTGCTGCTGTGTTCACA-3´_ | _5´-TCCGATCCAGGTTTTTAATGT-3´_ | _ann. 60 °C, mt 85 °C_ |

**Table S1**
